# Dicer regulates activation of the NLRP3 inflammasome

**DOI:** 10.1371/journal.pone.0215689

**Published:** 2019-04-23

**Authors:** David M. Ojcius, Ardavan Jafari, Laxmi Yeruva, Christian W. Schindler, Ali A. Abdul-Sater

**Affiliations:** 1 Department of Biomedical Sciences, Arthur Dugoni School of Dentistry, University of the Pacific, San Francisco, CA, United States of America; 2 School of Kinesiology and Health Science, Muscle Health Research Centre (MHRC), Faculty of Health, York University, Toronto, ON, Canada; 3 Department of Pediatrics, Arkansas Children’s Nutrition Center, University of Arkansas for Medical Sciences and Arkansas Children's Hospital Research Institute, Little Rock, AR, United States of America; 4 Department of Microbiology and Immunology, College of Physicians and Surgeons, Columbia University, New York, NY, United States of America; Shanghai Medical College, Fudan University, CHINA

## Abstract

Inflammation plays a critical role in initiation of adaptive immunity, pathogen clearance and tissue repair. Interleukin (IL)-1β is a potent pro-inflammatory cytokine and therefore its production is tightly regulated: its secretion requires the assembly of a macromolecular protein complex, termed the inflammasome. Aberrant activation of the inflammasome has been linked to debilitating human diseases including chronic inflammatory and autoimmune diseases. Thus, there is a great interest in understanding how inflammasomes are regulated. Here we show that Dicer, an enzyme necessary for the production of mature micro-RNAs (miRNAs), is required for optimal activation of NLRP3 inflammasomes in bone marrow macrophages. Our data indicate that miRNAs may play an important role in promoting inflammasome activation.

## Introduction

The innate immune system employs germ line encoded pattern recognition receptors (PRRs) that recognize “molecular patterns” from microbes, environmental stressors and damaged tissue. Once activated, these receptors initiate robust inflammatory responses that include the secretion of potent inflammatory cytokines such as Interleukin (IL)-1β, IL-6, TNF-α and Interferons (IFNs) [1–3]. IL-1β and IL-18 are among the most potent cytokines produced by human and murine innate immune systems, and they are released primarily as a part of the immune response to invading pathogens. However, excessive release of these cytokines, particularly IL-1β, is associated with autoinflammatory and autoimmune disorders [2–4]. Therefore, its secretion is tightly regulated, and typically requires two independent signals to be secreted. As with other cytokines, the first signal entails transcription of the IL-1β gene, but the gene product, pro-IL1β is not active. The second signal entails activation of the inflammasome, a multimeric protein complex typically composed of an NLR protein, the adapter protein ASC and the inactive zymogen caspase-1. Following inflammasome assembly, caspase-1 is activated and directs the cleavage of pro-IL1β into active and secreted IL-1β.

The canonical inflammasome can be nucleated by the NLR members NLRP1, NLRP3 and NLRC4 or by the ALR member AIM2. The NLRP3 inflammasome stands out among the inflammasomes for the large number of “danger associated molecular patterns (DAMPs)” it responds to and the large number of human diseases in which it has been implicated (reviewed in [5–7]). The DAMPs include molecules associated with cellular damage or sterile inflammation (e.g., ATP and uric acid), as well as molecules associated with metabolic diseases (e.g., elevated glucose, β-amyloid and cholesterol crystals), environmental stressors (e.g., silica, hydroxyl-apatite and asbestos crystals), as well as responding to pathogen associated toxins, like nigericin [8–11]). Gain-of-function mutations in the NLRP3 gene lead to autoinflammatory syndromes known collectively as cryopyrin-associated periodic syndromes (CAPS; reviewed in [12]). Importantly, dysfunctional NLRP3 inflammasomes are drivers of autoimmune diseases such as gout, rheumatoid arthritis (RA) and lupus [5, 6]. Moreover, chronic over-activation of the NLRP3 inflammasome is involved in the pathogenesis of debilitating metabolic disorders (e.g. obesity and atherosclerosis) and can also exacerbate liver damage (reviewed in [6]). For these reasons, cells employ several mechanisms to limit aberrant inflammatory responses to sterile insults and to cell death. This includes transcriptional regulation of the ASC and NLRP3 genes [10, 11]. Additional conserved negative regulators of inflammasome activity include MEFV (sequesters ASC; [13, 14]), Serpin B2, Serpin B9 (which inhibits caspase-1 proteolysis; [15]), PSTPIP1 (which interacts with MEFV; [16]) and NLRP10 (reviewed in [11]). Humans feature two additional families of negative regulators: pyrin only proteins (POPs) and CARD-only proteins (COPs; [10, 11, 17–20]).

MicroRNAs (miRNAs) are an evolutionarily conserved set of noncoding RNAs that fine-tune important physiological responses by targeting critical genes for down regulation (reviewed in [21–23]). These miRNAs are processed from a primary transcript by Drosha, a conserved RNAse III enzyme, into ~70 bp hairpin pre-microRNAs. Pre-microRNAs are exported to the cytoplasm, where they undergo additional processing by Dicer, a well-conserved RNAse III endoribonuclease, into 18–24 bp dsRNA. Dicer then facilitates loading of the antisense strand onto the RNA induced silencing complex (RISC). This complex is directed to complementary sequences in the 3’ UTR of target genes, culminating in their suppression [24]. miRNAs regulate an increasing number of important cellular processes, including apoptosis, cell proliferation and inflammation (reviewed in [21–23]). Consistent with this, dysregulation of miRNAs has been linked to a number of human diseases, including cancer and inflammation, RA and diabetes (reviewed in [22, 25]). More recently, several miRNAs have been implicated in regulation of signal 1 for inflammasome activation. Both miR-146a and miR-21, through their capacity to negatively regulate TLR or RIG-I signaling pathways, potentially impede the inflammasome Signal 1 [22, 26, 27]. In contrast, miR-155 has been reported to enhance Signal 1 by targeting two negative regulators, SHIP1 and SOCS1 [22].

In this report, we show that Dicer-deficient macrophages are significantly impaired in their ability to activate the NLRP3, but not the Aim2, inflammasome. Moreover, we demonstrate that certain miRNAs that are predicted to target negative regulators of the NLRP3 inflammasome are upregulated following inflammasome activation. These results suggest that miRNAs have an overall positive effect on NLRP3 inflammasome activation, potentially by targeting specific negative regulators of the inflammasome.

## Materials and methods

### Bone marrow macrophage preparation and inflammasome stimulation

Primary bone marrow-derived macrophages (BMMs) were prepared from Dicer fl/Cre- & Dicer fl/Cre+ mice, as we previously described [28]. Briefly, femurs and tibias were flushed with S+ media (RPMI 1640 supplemented with 10% Fetal Bovine Serum, non-essential amino acids, 2-mercaptoethanol, sodium pyruvate and penicillin-streptomycin), and RBCs were lysed with RBC lysis buffer before being plated into 100 mm Petri dishes containing S+ media containing 20% L929-conditioned media to drive their differentiation into macrophages. Cells were then treated with 1 μM 4-hydroxy tamoxifen (4-OHT; Sigma-Aldrich) at days 1, 5 and 6 to induce ERT-Cre mediated deletion of Dicer in cells from Dicer fl/Cre+. Day 7 BMMs were then primed with LPS (1 μg/ml, 4 h; *E*. *coli* type 025:86; Sigma-Aldrich) before stimulation with 5 mM ATP (1, 3 or 6 h; Sigma-Aldrich), or transfected (lipofectamine; Invitrogen) with 1 μg/ml Poly (dA:dT) (6 h; Sigma-Aldrich). For miRNA studies (see below), day 6 BMMs from WT C57/Bl6 mice were primed with LPS (1 μg/ml; 3 h) followed by stimulation with ATP (5 mM; 1 or 3 h) or nigericin (10 μM; 1 h) to activate the NLRP3 inflammasome.

No in vivo studies were performed. Prior to collection of bones, mice were euthanized by CO2 asphyxiation followed by cervical dislocation as recommended by the Panel on Euthanasia of the American Veterinary Medical Association. This animal protocol was approved by the Institutional Animal Care and Use Committee of Columbia University is (Approval number AAAD1804).

### Confirmation of Dicer deletion

Genomic DNA was prepared from BMMs of Dicer fl/Cre- & Dicer fl/Cre+ mice using DNeasy kit (Qiagen, Hilden, Germany), according to the manufacturer's instructions. PCR was then performed to confirm the deletion of the floxed allele in the Dicer fl/Cre+, as previously described [29].

### Immunoblotting

Proteins were obtained from supernatants of inflammasome-stimulated BMMs by trichloroacetic acid (TCA) precipitation (10% TCA for 10 min on ice). Pellets were dissolved in 2x Laemmli buffer and pH was readjusted with 1 M NaOH. Samples were then loaded onto 12% gels followed by SDS-PAGE and transferred to 0.2 μm PVDF membranes. The level of IL-1β and caspase-1 in culture supernatants was measured by immunoblotting with anti-IL-1β (AF-401-NA; R&D System; Minneapolis, MN) or anti-murine-caspase1 (SC-514; Santa Cruz; Santa Cruz, CA).

### Identification of potential miRNA targets by bioinformatic analysis

The miRNA target prediction was performed using the miRDB tool. mirDB employs a prediction program based on support vector machines (SVMs) and high-throughput training datasets. Targets in Table 1 were selected based on the “Target prediction score”, which is a new computational target prediction algorithm [30, 31]. Predicted targets with prediction score <60 were excluded because they indicate a low confidence and would require additional supporting evidence.

**Table 1:** miRNAs predicted to target some of the known regulators of inflammasomes. Information obtained from miRDB, an miRNA target prediction and functional annotation database, which uses the bioinformatics tool, MirTarget. Target scores below 60 were excluded from the analysis as they indicate low confidence in the prediction tool.

MiRNA	Target Gene	Inflammasome Component Regulated by Target Gene
mmu-miR-6951-3p mmu-miR-7116-3p	BRCC3	NLRP3
	BAD	Caspase-1, NLRP1 and NLRP3
	NR0B2	NLRP3
	SERPINB2	NLRP3 and ASC
mmu-miR-7078-3p mmu-miR-7056-5p	BRCC3	NLRP3
	EIF2AK2	NLRP3, NLRP1, NLRC4 and AIM2
mmu-miR-669n	BRCC3	NLRP3
	GBP5	NLRP3 and AIM2
mmu-miR-7210-3p mmu-miR-6929-5p mmu-miR-6344 mmu-miR-383-5p mmu-miR-504-3p	BRCC3	NLRP3
	SERPINB2	NLRP3 and ASC
mmu-miR-590-3p	POP1	ASC
	GBP5	NLRP3 and AIM2
	SERPINB2	NLRP3 and ASC
mmu-miR-344-5p mmu-miR-1964-5p	MEFV	NLRP3
	SERPINB2	NLRP3 and ASC
mmu-miR-126b-5p	MEFV	NLRP3
	NR0B2	NLRP3
mmu-miR-335-3p	BRCC3	NLRP3
mmu-miR-3058-3p		
mmu-miR-7681-5p		
mmu-miR-3075-5p	EIF2AK2	NLRP3, NLRP1, NLRC4 and AIM2
mmu-miR-3090-5p		
mmu-miR-6933-3p		
mmu-miR-7053-3p	GBP5	NLRP3 and AIM2
mmu-miR-6976-3p		
mmu-miR-7229-3p		
mmu-miR-6971-3p	MEFV	NLRP3
mmu-miR-6966-3p		
mmu-miR-9769-5p		
mmu-miR-5621-5p	NR0B2	NLRP3
mmu-miR-7033-5p		
mmu-let-7g-3p		
mmu-miR-879-5p	SERPINB2	NLRP3 and ASC
mmu-miR-7a-1-3p		
mmu-miR-7011-3p		

### miRNA isolation and real-time Q-PCR

Following NLRP3 inflammasome activation (see above), day 6 bone-marrow macrophages (BMM) from WT C57/Bl6 mice were washed with 1X PBS, and miRNAs were extracted by Pure Link miRNA Isolation Kit (Invitrogen; Carlsbad, CA) following manufacturer’s instructions. One μg miRNA was then converted into cDNA by miScript ll Reverse Transcription Kit (Qiagen, Hilden, Germany). Quantitative real-time PCR was performed with miScript SYBR Green PCR Kit (Qiagen). The Q-PCR reaction contained the universal reverse primer from the Qiagen kit, miRNA-specific forward primers (miScript Primer Assay; Qiagen) for ubiquitously expressed U6 small nuclear RNA (for normalization), mmu-miR-590-3p, mmu-miR-344, mmu-miR-6951-3p and mmu-miR-7078-3p along with 1:10 dilution of cDNA. Real time PCR was performed with an initial activation at 95°C for 15 min, followed by 40 cycles of 94°C for 15 s, 55°C for 30 s and 70°C for 30s on a Bio-Rad CFX384 Touch Real-Time PCR Detection System.

### Statistical analysis

Statistical analysis was carried out using GraphPad software (Prism) using student’s t-test, where P <0.05 was considered significant.

## Results and discussion

**Fig 1:**
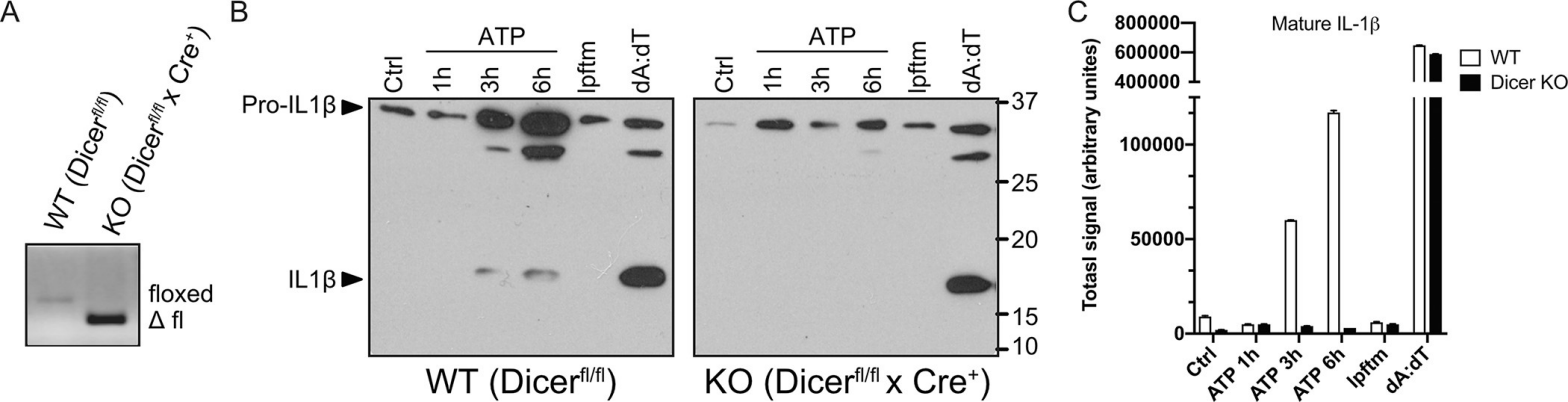
Dicer is required for optimal secretion of mature IL-1β following stimulation of the NLRP3 but not the AIM2 inflammasome. (**A**) Deletion of the floxed allele from day 7 bone marrow macrophages prepared from Dicer^fl/fl^ x ER^T^-Cre^-^ (WT) or Dicer^fl/fl^ x ER^T^-Cre^+^ (KO) mice was confirmed by PCR. (**B**) Dicerfl/fl x ERT-Cre- (WT) or Dicerfl/fl x ERT-Cre+ (KO) BMMs were primed with 1 μg/ml LPS for 4 h (signal 1) before stimulation with 5 mM ATP for 1, 3 or 6 hours or transfected (lipofectamine; lpftm) with 1 μg/ml poly dA:dT (dA:dT) for 6 hours (signal 2). Secreted IL-1β levels were then evaluated by immunoblotting. Arrows indicate the immature form of IL-1β (pro-IL1β) and the mature secreted form (IL-1β). (**C**) Quantitative analysis of the mature secreted form of IL-1β shown in panel B.

**Fig 2:**
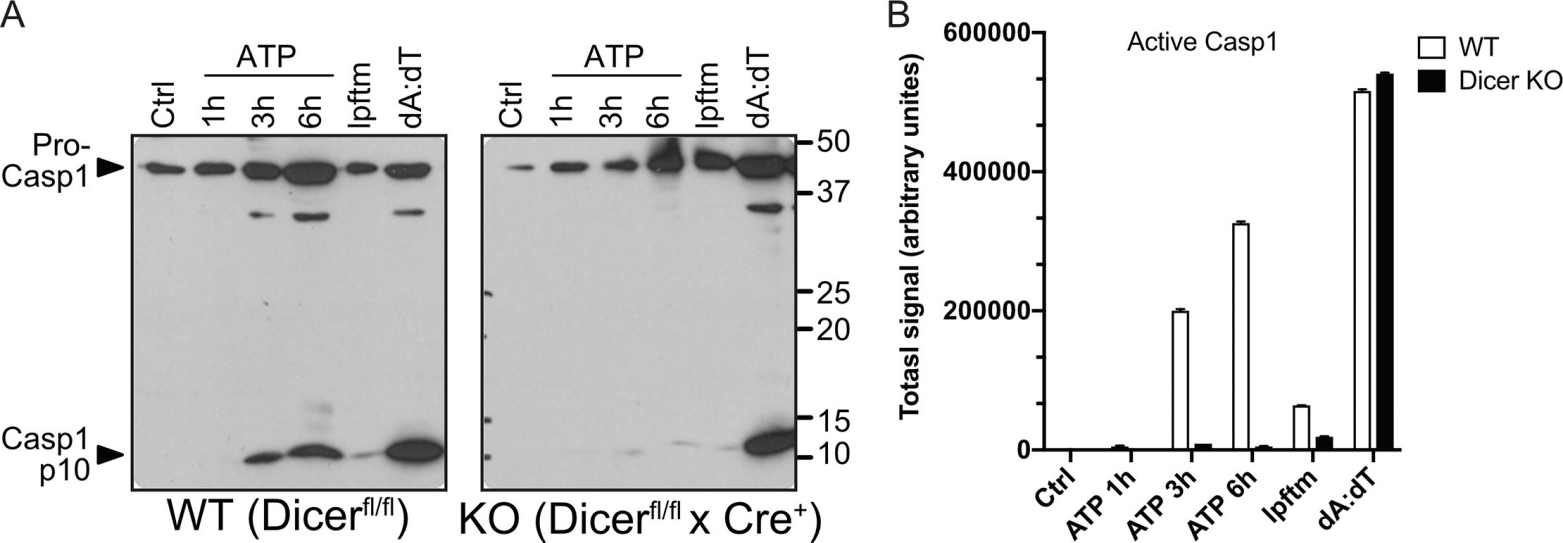
Dicer is required for activation of caspase-1 following stimulation of the NLRP3 but not the AIM2 inflammasome. (**A**) Day 7 bone marrow macrophages prepared from Dicer^fl/fl^ x ER^T^-Cre^-^ (WT) or Dicer^fl/fl^ x ER^T^-Cre^+^ (KO) mice were primed with 1 μg/ml LPS for 4 h (signal 1) before stimulation with 5 mM ATP for 1, 3 or 6 hours or transfected (lipofectamine; lpftm) with 1 μg/ml poly dA:dT (dA:dT) for 6 hours (signal 2). Secreted caspase-1 (casp1) levels were then evaluated by immunoblotting. Arrows indicate the inactive caspase-1 zymogen (pro-casp1) and the active form (casp1 p10). (**B**) Quantitative analysis of the mature secreted form of IL-1β shown in panel A.

Given the significance of inflammasome regulation in controlling inflammation and associated human diseases, we explored whether microRNAs under the control of Dicer play a role in NLRP3 inflammasome activation. Bone marrow macrophages (BMMs) were prepared from Dicer^fl/fl^ x ER^T^-Cre+ (*germ line deletion of Dicer is embryonic lethal*) and littermate control Dicer^fl/fl^ x ER^T^-Cre- mice. Addition of tamoxifen led to the efficient deletion of Dicer in the Cre+ but not Cre- cells as evident by the deletion of the floxed allele (Fig 1A; [29, 32]). As expected, stimulation of WT control, LPS-pretreated (*Signal 1*) Dicer^fl/fl^ x ER^T^-Cre^-^ macrophages with two well-characterized NLRP3 activators, ATP and nigericin, led to a robust increase in the secretion of processed IL-1β p17 (Fig 1B and 1C) and active caspase-1 p10 (Fig 2) into culture supernatants. Stimulation with poly(dA:dT), an AIM2 inducer, yielded an analogous increase in secreted IL-1β p17 and caspase-1 p10 (Figs 1B, 1C and 2). In stark contrast, there was a marked reduction in the secretion of both IL-1β and caspase-1 in ATP- and nigericin-stimulated Dicer-deficient macrophages (i.e., from Tamoxifen treated Dicer^fl/fl^ x ER^T^-Cre+ mice) (Figs 1B, 1C and 2). Remarkably, the response to poly(dA:dT) was not affected by the loss of Dicer. These results demonstrate that in the absence of Dicer, NLRP3 inflammasome but not AIM2 inflammasome activation is significantly impaired. Importantly, the ability of poly(dA:dT) to activate the AIM2 inflammasome in Dicer deficient macrophages provided strong evidence that ASC levels were not affected by Dicer deletion.

**Fig 3:**
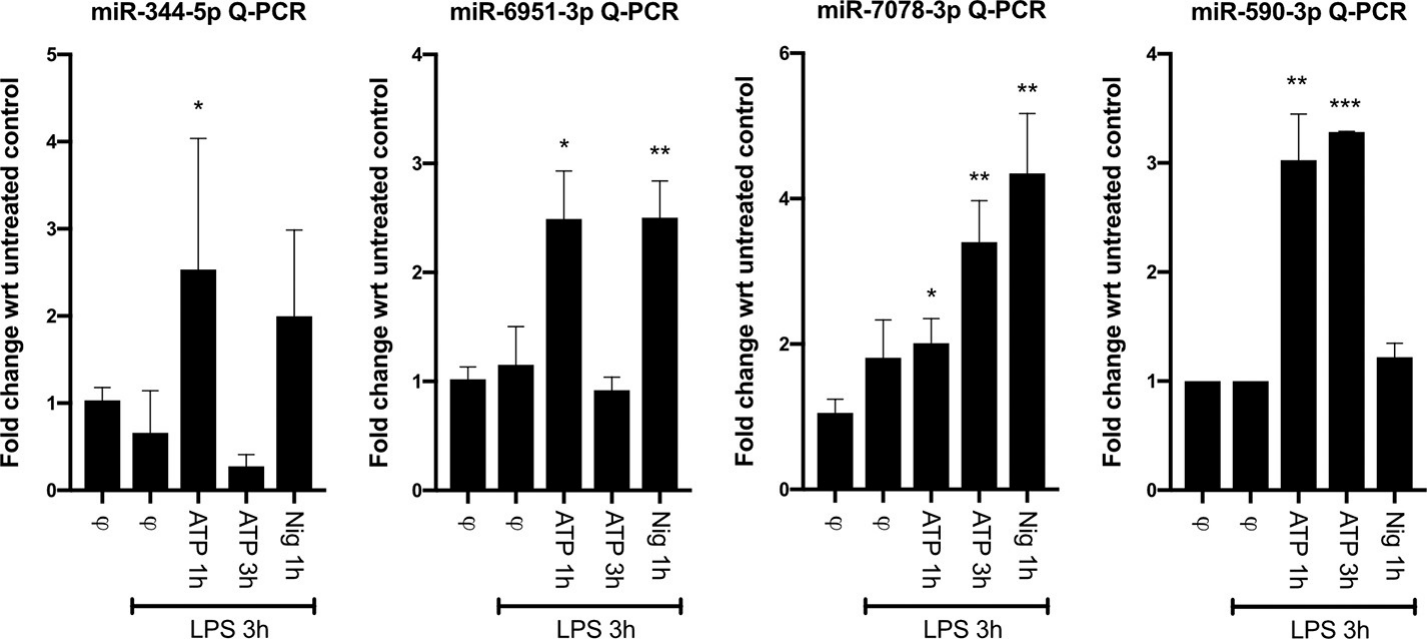
miRNAs predicted to target negative regulators of NLRP3 inflammasome are upregulated following inflammasome activation. Day 6 bone marrow macrophages prepared from WT C57/Bl6 mice were primed with 1 μg/ml LPS for 3 h (signal 1) before stimulation with NLRP3 inflammasome activators: 5 mM ATP for 1 or 3 hours or 10 μM nigericin for 1 hour (signal 2). Real-time Q-PCR analysis for the expression of mature miR-590-3p, miR-344, miR-6951-3p and miR-7078-3p was performed. Ubiquitously expressed U6 small nuclear RNA was used as a reference to normalize the data. * p<0.05; ** p<0.01; *** p<0.001.

One possible reason why miRNA deficiency due to the loss of Dicer could attenuate NLRP3 inflammasome activation is that some miRNAs might be required to relieve the inhibition mediated by certain negative regulators of NLRP3 inflammasomes. Thus, we employed miRNA target prediction databases to identify miRNAs that might target known negative regulators of the NLRP3 inflammasome but not the AIM2 inflammasome. The miRNAs that are predicted to target regulators of inflammasomes are shown in Table 1. In order to validate whether some of these miRNAs might indeed be involved in promoting inflammasome activation, we hypothesized that their expression levels should be upregulated upon inflammasome stimulation. Indeed, real-time Q-PCR analysis showed that mature miR-590-3p, miR-344, miR-6951-3p and miR-7078-3p levels are induced following activation of the NLRP3 inflammasome (Fig 3). Interestingly, the expression of these miRNAs was not induced by LPS stimulation alone (signal 1) and required signal 2 (ATP or nigericin), indicating that their role might be more important in sustaining NLRP3 inflammasome activity rather than for the initial activation. Interestingly, these miRNAs were induced with distinct kinetics (Fig 3). miR-344, miR-6951-3p peaked 1 h after ATP stimulation, while miR-7078-3p and miR-590-3p peaked 3 h after stimulation. This might be due to differences in the kinetics through which the targets of these miRNAs act to downregulate or shut down the inflammasome. These miRNAs target various negative regulators of NLRP3 inflammasomes and their upregulation might be required to sustain the activation of the inflammasome.

## Concluding statement

In this study, we explored whether miRNAs may regulate inflammasome activity. Using Dicer-deficient murine macrophages, we demonstrated, in the absence of miRNAs, a striking reduction in NLRP3 inflammasome activity, highlighting an overall positive role for miRNAs in this response.

Our observations reveal that overall, miRNAs play an important role in positively regulating the NLRP3, but not the AIM2 inflammasome. This positive regulation appears to be more important than or outweighs the negative activity recently attributed to miR-17, miR-21, miR-146a, and miR-223 [11, 22, 33–36]. Specifically, miR-223, whose expression is down regulated by lipopolysaccharide (LPS), was reported to suppress NLRP3 expression, and miR-17 has been implicated in the negative regulation of TXNIP, which may direct ER-stress dependent NLRP3 inflammasome activation [33–36]. Moreover, the positive regulatory activity observed by us does not appear to be secondary to miR-155, since there were no obvious defects in Signal #1. None of the predicted targets that could regulate inflammasomes shown in Table 1 have been described until now. Importantly, we show that some of these miRNAs are upregulated following activation of the NLRP3 inflammasome. Thus, the intriguing mechanism by which miRNAs potently upregulate the NLRP3 inflammasome will be the focus of future studies.
